# 

**Published:** 1907-11-15

**Authors:** 


CONTENTS

Event and Comment - - - - - - - - 539

Pressure Anesthesia, - - - *By Charles C. Hampton, D.D.S.* 568

Indents - - - - - - - - - - - 590

Announcements - - - - - - - - - 591

